# The reduction in relative brain size in the domesticated dog is not an evolutionary singularity among the canids

**DOI:** 10.1098/rsbl.2024.0336

**Published:** 2024-08-05

**Authors:** László Zsolt Garamszegi, Niclas Kolm

**Affiliations:** ^1^ Institute of Ecology and Botany, HUN-REN Centre for Ecological Research, Vácrátót, Hungary; ^2^ Department of Zoology, Stockholm University, Stockholm, Sweden

**Keywords:** brain size, cognition, dogs, evolutionary singularity, hibernation, phylogenetic comparative methods

## Abstract

Domestication has long been considered the most powerful evolutionary engine behind dramatic reductions in brain size in several taxa, and the dog (*Canis familiaris*) is considered as a typical example that shows a substantial decrease in brain size relative to its ancestor, the grey wolf (*Canis lupus*). However, to make the case for exceptional evolution of reduced brain size under domestication requires an interspecific approach in a phylogenetic context that can quantify the extent by which domestication reduces brain size in comparison to closely related non-domesticated species responding to different selection factors in the wild. Here, we used a phylogenetic method to identify evolutionary singularities to test if the domesticated dog stands out in terms of relative brain size from other species of canids. We found that the dog does not present unambiguous signature of evolutionary singularity with regard to its small brain size, as the results were sensitive to the considerations about the ancestral trait values upon domestication. However, we obtained strong evidence for the hibernating common raccoon dog (*Nyctereutes procyonoides*) being an evolutionary outlier for its brain size. Therefore, domestication is not necessarily an exceptional case concerning evolutionary reductions in brain size in an interspecific perspective.

## 1. Introduction

Domestication is often highlighted to be the most important factor behind dramatic negative effects on brain size—that is positively associated with cognitive abilities—when domesticated animals are compared with their wild conspecifics [1–6]. The prevailing idea behind this pattern is that domestication provides relaxed selection from selection pressures such as finding food, finding mates and avoiding predation, and this shifts the balance in necessary investment into metabolically costly brain tissue [7]. However, a recent review of existing evidence from different species calls for careful interpretations, because trait variation in recent domestic animal breeds may not reflect changes that occurred during domestication [8].

Analyses of domestication effects on brain size are most often comparing domesticated breeds to their single ancestral species [1–6]. To investigate if domestication is a particularly powerful agent behind patterns of decreased brain size, it would be effective to also compare brain size of domesticated species including their potential sub-breeds with other closely related species. This would be helpful for a correct evolutionary interpretation of domestication, given that domestication is often regarded as an exceptional case. Here, we provide such an analysis across the Canidae group.

Canids are a group of carnivores that is represented by 14 genera and 34 species. They are considered to be medium sized among the carnivores [9–11]. The dog is a canid species that was domesticated from its ancestral species the grey wolf at least 15 000 years ago [12]. Over 400 breeds of dogs exist today, and the overall pattern is that relative brain size is dramatically reduced in dogs (>24% reduction) as compared with the ancestral species, the grey wolf [5]. The dog is considered to be the first domesticated species, which makes it highly suitable as a model for testing if the domestication effect on brain size is particularly powerful, or if other non-domesticated species of canids display similarly small brains.

Here we use a phylogenetic comparative method that allows for identification of evolutionary singularities [13] to investigate if the dog stands out in terms of relative brain size in comparison with other canid species. The principal idea behind this approach is that if an exceptional character state occurs along a particular branch of the phylogeny, this should result in an observed phenotypic value that does not correspond with what the pattern of co-evolution of traits would predict along that particular branch. We predict that dog relative brain size should be identified as an evolutionary singularity if domestication is particularly important behind reductions in relative brain size.

## 2. Methods

### (a) Data assembly

Data on brain size (in the form of volume) and body size (in the form of mass) for 25 canid species were obtained from the literature. For wild canids, we relied on information presented in Tsuboi *et al.* [14], from which we checked the primary references for the original data. Furthermore, we updated these with data from more recent sources (electronic supplementary material, table S1). Data on the dog were derived from Garamszegi *et al.* [5]. We also used data from the literature for the dingo (*Canis familiaris dingo* [15]) and for the extinct dire wolf (*Aenocyon dirus* [16,17]). Some studies reported brain mass, thus we transformed these into brain volume data using the equation of the regression line fitted to the observed data for species with information on both measures of brain size: log_10_(brain volume) = −0.102 + 1.053 * log_10_(brain mass) [5].

There is a great variation in both brain size and body size among the modern breeds of dogs, and this variation does not necessarily reflect trait expression values when domestication occurred. Unfortunately, we were unable to locate comparable data for the traits of interest for an ancestral dog breed, thus we used information on body mass and brain volume from ancient dog breeds to approximate a state that is at least closer to the dogs’ ancestor. Ancient breeds that originate more than 500 years ago show genetic admixture with wolves and represent an early stage of dog domestication (e.g. [5, 18–20]). Hence, we included 11 typical ancient breeds for which we had data (Greenland sled dog, basenji, akita inu, Saluki, Siberian husky, Afghan hound, Samoyed, Alaskan Malamute, Chinese shar pei, chow chow and greyhound). To appreciate the variance of traits that can be observed among the ancient breeds, we performed the analysis by considering each of them one by one to reflect trait values of the dog during domestication and interpreted these separate results jointly for a general conclusion. To help visual interpretations, we present figures that consider the main trait values for the dog over the 11 ancient breeds.

In addition, extant wolves are not the direct descendants of the wolves that gave rise to dogs, while they can also be characterized by considerable among-population differences. In this study, we combined information from nine independent sources representing samples from different parts of the world and calculated mean body mass and brain volume across more than 100 individuals. Given that we had multiple data for other species as well (see electronic supplementary material, table S1), we could calculate the within-species repeatability of traits (*sensu* [21]). These estimated values were convincingly high (body mass: *R* = 0.883, CI = [0.809, 0.935], brain mass: *R* = 0.951, CI = [0.863, 0.979], brain volume: *R* = 0.573, CI = [0.318, 0.761]) to conclude that within-species variation in the wolf (and in other wild species) is negligible for the evolutionary hypothesis investigated in the given among-species context. To deal with the uncertainty of the ancestral wolf, from which the dogs were originated, we included data for two other reference species around the split of the wolf/dog lineage on the phylogenetic tree (the dingo and the dire wolf). These outgroups do not represent the ancestor of dogs either, but their inclusion introduces a considerable variance for the possible trait values of the ancestral state. Note that this study does not aim to contrast relative brain size in the dog with relative brain size of the wolf ancestor specifically, but aims to investigate observed trait expression in comparison with the among-species distribution in general, and by considering the whole phylogeny of canid species. In this respect, the uncertainty of the origin of the dog cannot be a serious confounder.

Phylogenetic relationships among canid species were reconstructed based on Bininda-Emonds *et al*. [22], to which the tip for the dog was added as close relative to the grey wolf. We also added the dingo as a close relative to the domestic dog, and the extinct dire wolf as a sister species to the wolf to provide more contrasts around the origin of the dog (if we remove the dingo and/or the dire wolf from the analyses, the results presented below remain qualitatively unchanged). The phylogenetic tree included polytomies (electronic supplementary material, figure S1), which were randomly resolved 100 times. These resolution repeats were stored as a set of different possible phylogenetic trees and were subsequently used in the phylogenetic comparative model to take into the account the uncertainty due to the non-bifurcating nodes in the phylogeny.

### (b) Statistical analyses

To understand the effect of domestication in an interspecific phylogenetic context, we investigated the presence of evolutionary singularities along the allometric relationship between brain size and body size in canids by using the phylogenetic prediction approach [23–25]. This framework is straightforward when one has an *a priori* expectation of a singularity in a given trait along a given lineage and relies on a formal outlier test that can be performed based on the functions ‘BayesModelS’ that was written by Nunn & Zhu [13] in the R statistical environment [26]. ‘BayesModelS’ generates distributions of predicted trait values for the species of interest given a list of predictor variables and the phylogeny considered in a phylogenetic generalized least squares (PGLS) modelling framework. It follows a Markov chain Monte Carlo (MCMC) algorithm to fit parameters by assuming a Brownian motion model of evolutionary change [27] and also allows for accounting of phylogenetic uncertainty by sampling from a set of trees [28] and adjusting for the branch length scaling factors. For more details about the ‘BayesModelS’ functions, Nunn & Zhu [13], for which the source code is also available (https://github.com/MPCMEvolution/MPCMArchive/wiki/21.-Chapter-21).

To investigate if the relatively small relative brain size in dogs due to domestication can be considered an evolutionary singularity, we made predictions for the phenotypic expression of the trait in this particular species. For this, we first built a PGLS model to define the allometric scaling of brain size across canid species, in which the dog’s data were not included to avoid biasing the predictions. Then, based on the estimated parameters of the PGLS model, we generated a posterior probability distribution of predicted brain size in the dog. This posterior distribution was thus defined by considering the dog’s body mass, the estimated trait co-variation, and phylogenetic signal in the data. We finally examined if the observed brain size in the dog differs from the predicted distribution. A species can be identified as an outlier when its observed trait expression falls outside the 95% credible interval of the predicted posterior probability distribution. We repeated this procedure for each ancient dog breed representing the ancestral state during domestication.

We used the following parameter setting in ‘BayesModelS’: burn-in, 100 iterations; posterior sampling, 200 000 iterations (after burn-in); thinning interval, 100 iterations (resulting in 2000 samples for the posterior distribution). The response variable was log_10_(brain volume), and the predictor variable was log_10_(body mass). The MCMC chain sampled the set of 100 phylogenies that were generated by the random resolution of polytomies. We set the lambda branch length scaling parameters to be estimated. Before interpreting the model outcomes, we performed the necessary model checking diagnostics to verify the that MCMC was appropriately converged [13].

## 3. Results

**Figure 1.**
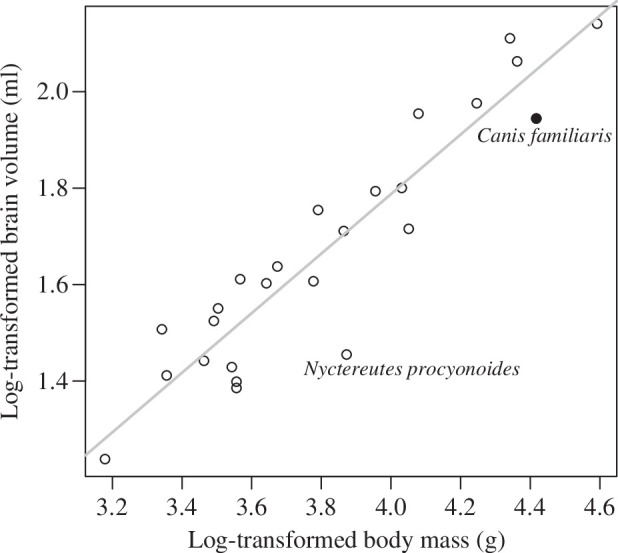
Brain–body evolutionary allometry in canids. Points are species-specific estimates of trait values and the line is the least square regression line fitted on them. The domesticated dog is highlighted with a filled point (that shows the mean trait values across 11 ancient breeds). The data point (reflecting the mean trait values across 52 individuals of mixed sexes) for the common raccoon dog, which is the only canid species that hibernates, is labelled with its scientific name and shows that this species has the smallest brain size as expected from its body size.

The allometric regression of brain size for the canid species is shown in figure 1. The distribution of points around the fitted slope suggests that the small brain size of dogs relative to their body size is not an exceptional case. The residual for this species is −0.101, and there are seven other canid species that can be characterized with larger absolute residual value.

**Figure 2.**
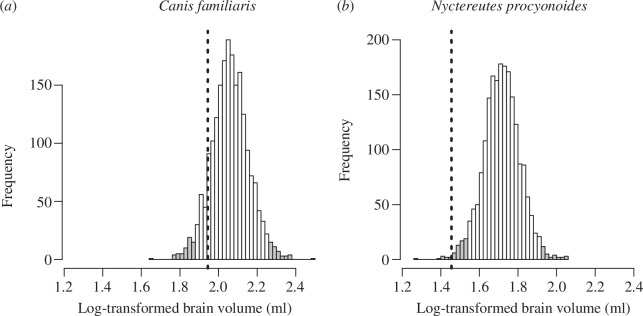
The posterior distributions of predicted brain size values generated by ‘BayesModelS’ for the dog (*a*) and the common raccoon dog (*b*) with body mass used as the predictor variable. For simplicity, the underlying analyses consider the average trait values for 11 ancient dog breeds to reflect brain and body size during the domestication (for models performed separately based on different ancient breeds see table 1). Vertical lines indicate observed values and shaded parts of the distributions show the ranges that fall outside the 95% credible intervals.

We investigated if the observed brain volume in ancient dogs can be considered as evolutionary singularity in canids. The phylogenetic predictions revealed that the observed mean value was included within the 95% credible interval of the posterior distribution of the predicted values for eight out of the 11 runs based on different breeds used to reflect the ancestral during domestication (table 1). On average, out of the 2000 sample, 378 predictions (18.89%) were smaller than the observed value (figure 2*a*). However, when using data from akita inu, Alaskan Malamute and chow chow, we derived significant evidence for relative brain size in the dog being an evolutionary singularity.

**Table 1.** The outcomes of tests of evolutionary singularity when considering different ancient breeds separately to reflect trait values during domestication. Values of *p* indicate whether observed brain size of the given breed was included within the 95% credible interval of the posterior distribution of the predicted values (i.e. the proportions of predictions that were smaller than the observed value in the 2000 sample). Lambda values provide the means of estimates from the Bayesian posterior distribution of phylogenetic models.

breed	body mass (g)	brain volume (ml)	*P*	lambda
Greenland sled dog	31 000	64.97	0.112	0.409
basenji	10 000	54.88	0.233	0.400
akita inu	44 800	106.79	0.011	0.399
Saluki	22 500	87.65	0.203	0.407
Siberian husky	22 954	90.33	0.229	0.408
Afghan hound	22 800	97.24	0.347	0.426
Samoyed	22 250	90.39	0.331	0.414
Alaskan Malamute	36 900	113.94	0.040	0.407
Chinese shar pei	16 900	83.96	0.406	0.404
chow chow	25 667	74.52	0.026	0.414
greyhound	31 842	103.13	0.143	0.409

Visual inspection of the species-specific data around the allometric regression (figure 1) revealed that the common racoon dog (*Nyctereutes procyonoides*) might be an outlier. This species has the smallest residual (−0.254), which indicates a deviation from the regression line, which is more than 2.5 times larger than it can be observed for the dog. Therefore, we tested if this species represents a biologically relevant evolutionary singularity in the canid clade. The ‘BayesModelS’ analysis predicting brain size based on body mass while excluding data for the common racoon dog revealed that the observed values for this species can indeed be considered an evolutionary singularity (figure 2*b*). More than 99.6% of predictions fell above the observed values for brain size in the common raccoon dog.

## 4. Discussion

Contrary to our prediction, the dog’s relative brain size did not stand out clearly as an evolutionary singularity in our analysis, and the results were sensitive to the considerations about the ancestral trait values upon domestication. This suggests that domestication is not necessarily a particularly powerful factor behind reductions in relative brain size and that other factors could be as important in an interspecific context when considering what selection pressures might lead to reductions in relative brain size. For example, antipredator defences such as body armour and spines have been shown to correlate with smaller brains [29]. Furthermore, selection for reduced brain size might be relaxed in dogs, given that during the domestication process they entered into mutual cooperation with humans with social relationships while also maintaining some degree of independence [30]. These factors may still require the maintenance of enhanced cognitive abilities contrary to other animals that were domesticated for simple economic exploitation (nutrients, fur or labour).

Our *post hoc* analysis identified the common raccoon dog as the only evolutionary singularity among the canids. Information on the specific ecology of a single species does not permit for general conclusions about what ecological factors might have led to the reduction in relative brain size in the common raccoon dog. However, we find it appealing that this species is the only species among the canids that hibernates [31,32]. Hibernation has been suggested to constrain brain size evolution, and a recent comparison of hibernators versus non-hibernators in mammalian species supports this idea [33]. The main mechanism believed to explain this pattern is that prolonged periods of food shortage, such as during hibernation, prevents the evolution of large brains due to their constantly high energy demands (see discussion on this topic in [33]).

The comparative method used here was previously used to identify the unusually large human brain relative to the body as an evolutionary singularity among hominines [27] and primates [13]. Using the same method, we did not find similarly strong support for dog domestication being an evolutionary singularity behind small relative brain size in canids. We propose that the role of domestication should not be overemphasized as a key factor mediating the evolution of small brains, and that a more balanced view of its role would be beneficial from an evolutionary point of view. We suggest that future studies should add other selection pressures when investigating reductions in brain size in relation to domestication and also that such analyses should test for evolutionary rates across the phylogeny, which could provide further insights on how brain size evolved in the domesticated dog in relation to other canid species. Such an analysis would also benefit from using archaeological data to reflect brain and body size for an ancient dog that lived around the time of domestication. Furthermore, it would be straightforward to perform similar analysis of evolutionary singularity in other animals that left behind a larger pack of cognitive skills upon domestication than the dog.

## Data Availability

All data used in the study will be available in the electronic supplementary material [34].
